# Adaptation and usability study of the fear of cancer recurrence therapy for parents of survivors of childhood cancer (Parent-FORT)

**DOI:** 10.3389/fpsyg.2026.1815418

**Published:** 2026-09-08

**Authors:** Celeste Holy, Jeffery Brinson, Janelle Gibson-Haerle, K. Brooke Russell, Lesleigh S. Abbott, Lauriane Giguère, Amy Givogue, Gregory M. T. Guilcher, Lauren C. Heathcote, Emily Johnson, Kimberly McMillan, Christine Maheu, Wendy Pelletier, Fiona Schulte, Héloïse Sirois-Leclerc, Perri R. Tutelman, Sophie Lebel

**Affiliations:** 1 School of Psychology, University of Ottawa, Ottawa, ON, Canada; 2 Parent Partner, Hamilton, ON, Canada; 3 Children’s Hospital of Eastern Ontario, Ottawa, ON, Canada; 4 Section of Pediatric Oncology/Cellular Therapy, Alberta Children’s Hospital, Cumming School of Medicine, University of Calgary, Calgary, AB, Canada; 5 Health Psychology Section, Institute of Psychiatry, Psychology, & Neuroscience, King’s College London, London, United Kingdom; 6 School of Nursing, Faculty of Health Sciences, University of Ottawa, Ottawa, ON, Canada; 7 Ingram School of Nursing, Faculty of Medicine and Health Sciences, McGill University & Research Institute, Montreal, QC, Canada; 8 Department of Oncology, Division of Psychosocial Oncology, Cumming School of Medicine, University of Calgary, Calgary, AB, Canada

**Keywords:** cancer, childhood neoplasms, fear of cancer recurrence (FCR), group psychotheraphy, intervention, parents, psychology, survivors

## Abstract

**Introduction:**

Fear of cancer recurrence (FCR) is a common and distressing concern for caregivers of survivors of cancer. At clinical levels, FCR is persistent, impairs quality of life, increases psychological distress, and increases health-system costs. Emerging evidence suggests that about half of parents of survivors of childhood cancer report clinically significant FCR, with associated negative impacts. No interventions currently target parent FCR. The Fear of Recurrence Therapy (FORT) group intervention efficaciously reduces FCR and improves quality of life for survivors of adult cancer. Our aim was to adapt FORT for virtual delivery for parents of survivors of childhood cancer and evaluate its usability.

**Methods:**

Using the ADAPT and FRAME frameworks, we collaborated with 10 parents with lived experience to modify FORT for this population. Following adaptation, two trained therapists facilitated a usability trial of the adapted program (Parent-FORT) with one group of parents. Therapists and participants completed feedback questionnaires after each session to assess usefulness, usability, and readiness for future users, as well as a final exit interview. Descriptive statistics were used to summarize questionnaire data. Content analysis was used to summarize qualitative data from exit interviews.

**Results:**

Five mothers whose children were diagnosed with childhood cancer were enrolled and four (Alberta, *n* = 2; Ontario, *n* = 2) completed the usability trial. Significant changes were made to the intervention language and content, resulting in an additional session. Key changes included the addition of exercises on navigating parent–child conversations about new physical symptoms and on exploring identity after cancer. Inclusion criteria were also broadened from one-year post-treatment to one-month post-treatment. Feedback indicated preliminary evidence of usability and acceptability. Qualitative analyses of exit interview data identified four themes: connectedness of the group, general format, satisfaction with the content and exercises, and overall impact.

**Discussion:**

Parent-FORT reflects the first FCR intervention designed specifically for parents of survivors of childhood cancer. Meaningful co-design with parents was essential to the success of the adaptation. These preliminary findings support Parent-FORT’s readiness for testing in a future feasibility pilot study.

## Introduction

Fear of cancer recurrence (FCR) is defined as the fear, worry, or concern that cancer may come back or progress (Lebel et al., 2016). Clinically significant FCR, defined as moderate-to-high levels of fear, affects approximately 50% of cancer survivor caregivers and tends to remain stable over time if unaddressed (Smith et al., 2022). It is associated with reduced quality of life (QOL), increased psychological distress, internalizing symptoms, and higher FCR for survivors themselves (Boehmer et al., 2016; Braun et al., 2021; Lin et al., 2018; Maheu and Galica, 2018; Smith et al., 2022; Webb et al., 2023). While most research in this domain has focused on caregivers of survivors, a recent systematic review and meta-analysis identified six studies examining FCR among parents of survivors of childhood cancer (Webb et al., 2023). Results of these studies demonstrated that higher FCR among parents was associated with reduced QOL and increased distress. Further, parents reported worse FCR than survivors themselves, with approximately half reporting clinically significant fear (Webb et al., 2023).

Evidence suggests that parents with higher FCR are more likely to contact their child’s medical team and schedule more medical appointments for their child (Tutelman et al., 2022). Thus, unaddressed FCR in parents has implications for the broader healthcare system (Tutelman and Heathcote, 2020). Parents with high levels of this fear have also reported greater communication difficulties with their children and reluctance to let their child take on increasing responsibility for managing their own illness (Murphy et al., 2021; Reed-Knight et al., 2014). This may in turn increase the risk that pediatric survivors themselves will develop FCR as they enter adolescence and young adulthood (Tutelman and Heathcote, 2020). FCR interventions for parents may therefore reduce this fear among parents while also helping to prevent its development in pediatric survivors.

While many psychosocial interventions have been developed to address the unique needs of parents of children with cancer, none have specifically targeted FCR (Ogez et al., 2019; Ong et al., 2025). Some interventions, such as CASCADE (Wakefield et al., 2021) and ENGAGE (Woodford et al., 2021), have included brief or indirect content on FCR, but evaluation of FCR outcomes have not demonstrated meaningful change. These findings are consistent with the broader adult literature, including evidence that a targeted FCR intervention was superior at reducing this concern compared to a structurally equivalent support group that discussed general survivorship topics (Maheu et al., 2023). Overall, these results suggest that interventions designed to address distress more broadly may not generalize to FCR. Instead, improving FCR may require more targeted intervention including psychoeducation about recurrence and FCR, opportunities to practice applying coping skills directly to recurrence-related fears (e.g., cognitive restructuring, exposure), and guidance on how to communicate with others about their fears.

Encouragingly, interventions directly aimed at reducing FCR in adult survivors have demonstrated strong efficacy (Maheu et al., 2023; Tomei et al., 2018). Among these interventions is the Fear Of Recurrence Therapy (FORT), a weekly, 6-session cognitive-existential therapy intervention (Lebel et al., 2014). FORT has demonstrated success in reducing clinical FCR as well as improving QOL and reducing intrusive thoughts, anxiety, and depression for adult survivors in two randomized controlled trials (Maheu et al., 2023; Tomei et al., 2018). Given its evidence base, manualized protocol, and successful implementation within Canadian healthcare systems (Lebel et al., 2025), FORT provides a strong foundation for adaptation.

We recently adapted FORT for family caregivers of adult survivors of cancer (FC-FORT) and demonstrated that FC-FORT is feasible for online delivery, positively evaluated by participants, and associated with reductions in clinical FCR (Lamarche et al., 2024). While promising, the unique context of childhood cancer and the developmental life stage of pediatric survivors suggest further adaptations of FORT are needed to ensure a good fit for this specific parent population. As such, the goals of this study were to (1) adapt FORT for parents of pediatric survivors of cancer, guided by published theory (Tutelman and Heathcote, 2020), research evidence, and an advisory board of parents of survivors of childhood cancer, clinicians, and researchers and (2) test its readiness for future evaluation via a usability study.

## Methods

### Intervention

#### The Fear of Recurrence Therapy (FORT)

FORT is a cognitive-existential group therapy intervention originally developed for survivors of adult-onset cancers. The original version consists of 6 consecutive weekly sessions of 120 min with weekly homework assignments (Maheu et al., 2023). Its therapeutic framework is informed by a blended FCR theoretical model developed and empirically validated through path analyses (Lebel et al., 2018). The key goals of FORT are to: (1) distinguish worrisome symptoms from benign ones; (2) identify FCR triggers and inappropriate coping strategies; (3) facilitate the learning and use of new coping strategies, such as relaxation techniques and cognitive restructuring; (4) increase tolerance for uncertainty; (5) promote emotional expression of specific fears that underlie FCR; and (6) re-examine life priorities and set realistic goals for the future. FORT is fully manualized, with manuals available for both patients and therapists.

### Procedures

#### The adaptation frameworks

**Figure 1:**
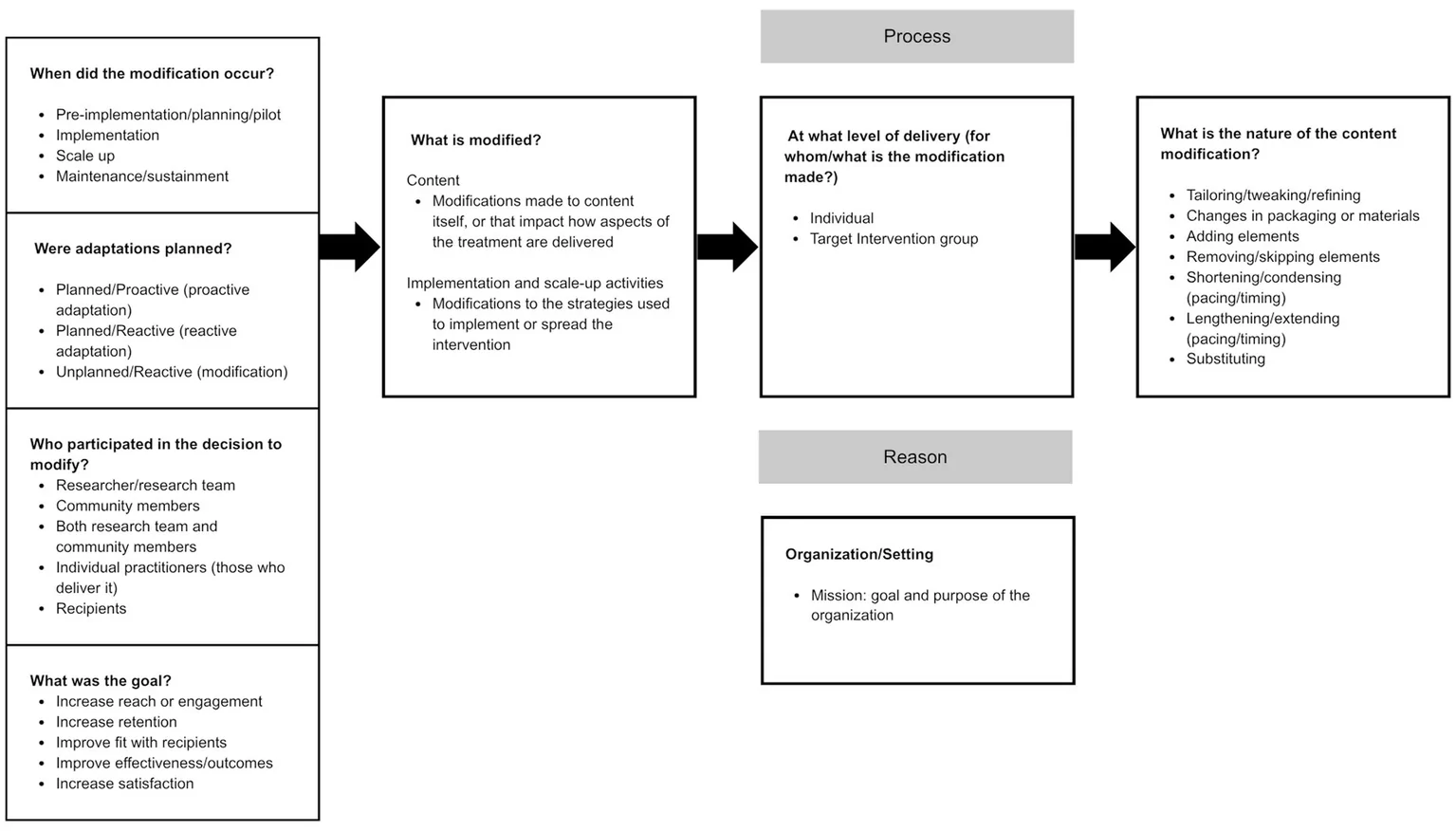
Modifications to the Parent-FORT intervention based on on the FRAME framework (Wiltsey Stirman et al., 2019).

We followed the ADAPT guidance (Moore et al., 2021) which proposes a systematic process for adapting evidence-based health interventions to new contexts to ensure a good fit. As service-user involvement is central to this framework, two parent partners (JG-H and JB) were included on the primary research team as parent co-chairs. Eight additional parents contributed to an advisory board (described below) during the initial adaptation phase of the overall project. Additionally, we adhered to the Framework for Reporting Adaptations and Modifications-Expanded (FRAME) (Wiltsey Stirman et al., 2019), which assists in describing: when and what modifications occurred, reasons and goals for modifications, whether they were planned or unplanned, who participated in the decision to modify, who the modifications were made for, and relationship to fidelity. Two team members (CH, SL) independently coded each modification in an Excel spreadsheet based on the FRAME framework categories, utilizing the FRAME coding manual (see Figure 1). The coders met to resolve any discrepancies until consensus was reached.

#### Adaptation team

For the initial adaptation, we assembled an adaptation team comprised of the research team (2 pediatric oncologists, 1 pediatric oncology social worker, 1 pediatric oncology clinical psychologist, 4 psychology and 2 nursing researchers, parent co-chairs who represented 1 mother and 1 father of Pediatric survivors of cancer, and a master’s level research coordinator) and the parent advisory board (7 mothers and 1 father of survivors of childhood cancer). Parents on the advisory board were recruited through a national call for expressions of interest. Parents each had one child (< 18 years of age) who had completed treatment for cancer at least one year prior. The parent advisory board was larger than anticipated to ensure members adequately represented Canadian parents and included a range of diversity factors. Of the 10 parents on the advisory board, eight were White and two were South Asian, one was a single parent, and two lived outside of major urban centers. Six were located in Ontario, three in Alberta, and one in British Columbia. At the time of diagnosis, five parents had children who were 8–10 years, three were 4–7, and two were 0–3. Time since active treatment ranged from 1 year to more than 8 years. We followed best practices in patient engagement according to the Canadian Institutes of Health Research Strategy for Patient-Oriented Research (Government of Canada, 2018) and compensated parents on the advisory board according to the CHILD-BRIGHT compensation guidelines (CHILD-BRIGHT, 2022). Terms of reference were shared with parents on the advisory board prior to their agreement to the role, which outlined the role description, meeting structure, timeline, and compensation.

#### Initial adaptation

The adaptation team met virtually eight times between January – April 2024 to oversee the initial adaptation of Parent-FORT. The virtual meetings were co-led by the two parent co-chairs (JG-H and JB) and a member of the research team (KM). Other research team attendees included a FORT expert (SL) to answer questions about the content of the sessions and the research coordinator (CH) for administrative purposes. To reduce potential power imbalance between researchers and patient partners, additional research team members did not attend the virtual meetings and were primarily consulted via email. Following an initial introductory meeting, each meeting was used to discuss adaptations for a single consecutive session of FORT. Several strategies were used to maximize input from the advisory board: (1) parents were asked to come prepared with their top three points of feedback for each session, which were shared in a roundtable format; (2) virtual meetings were scheduled in advance and occurred bi-weekly to make the time commitment more manageable and provide sufficient notice to do the assigned readings; (3) a parent who was not co-chair began the three takeaways to ensure all parents on the advisory board were equal participants. Co-chairs would guide the discussion when necessary to ensure healthy dialogue; and (4) co-chairs and members of the research team in attendance fostered a climate of psychological safety and encouraged members to voice their disagreement. The advisory board was also asked for their feedback regarding other aspects of the study (e.g., inclusion criteria, recruitment strategies, diversity considerations). Adaptation meetings were well attended by the advisory board, with between 7 to 10 (of 10) members at each meeting. When someone was unable to attend the meeting in person, they provided their feedback via email to ensure their perspectives were captured.

#### Usability study

Following the initial adaptation, we conducted a virtual usability study of Parent-FORT using the adapted manual to further refine the intervention. Our aim was to recruit 6 to 9 parents of pediatric survivors of cancer to participate in the Parent-FORT intervention, co-facilitated by two trained therapists. After each session, the therapists and participants completed short feedback questionnaires about the session. Participants who missed a session were offered one individual videoconference make-up session. Participants were informed during the consent process that if they missed more than one session, they would be required to withdraw and join the next group as too much content would have been missed to continue. At the end of the intervention, participants and therapists were also invited to complete a 1:1 virtual exit interview to discuss any further changes needed. Participants were compensated with a $20 gift card for completing the exit interview.

Readiness of the intervention for a future feasibility pilot study was evaluated using data from session feedback questionnaires, exit interviews, and consultation with parent partners and the broader research team. An additional usability study was planned if one or more sessions received a low overall rating or if identified areas for improvement required substantive changes (e.g., adding, moving, or removing modules). If suggested changes were minor (e.g., minimal changes to wording or examples), revisions were reviewed with the larger research team including parent co-chairs to determine whether further testing was warranted.

#### Psychotherapist training and supervision

We recruited and trained two licensed therapists to facilitate the sessions. The lead researcher (SL) provided therapists with a one-day virtual Parent-FORT training, including review of the clinician manual, followed by weekly 30-min virtual supervision to address any challenges and prepare for the next session. In addition, session recordings were reviewed and scored by the study coordinator (CH) using a fidelity checklist. If adherence was less than 80% on any session, the lead researcher (SL) provided additional feedback to the therapists running the usability study.

### Participants

#### Usability study participants

Inclusion criteria: (1) parent (or primary caregiver) of a survivor of childhood cancer who is under 18 years of age and at least 12 months from the completion of active treatment for any type of cancer; (2) a clinically significant score (13+) on the Fear of Cancer Recurrence Inventory-Parent version (Tutelman et al., 2022) (FCRI-P; range = 0–36); (3) access to a computer and internet connection; and (4) living in Canada. Only one parent/caregiver per child could participate in the study. Exclusion criteria: (1) Parents of children with active graft-versus-host disease; (2) non-English speakers; (3) Parents concurrently participating in another therapist-led psychosocial therapy group; and (4) Parents with an undermanaged mental health disorder judged to be clinically contra-indicated and/or likely to affect the group work, based on disclosure by the potential participant or clinically identified by the group therapist at pre-therapy interview (e.g., individuals with a severe major depressive episode requiring treatment prior to participation; individuals with stable, medically managed, or low severity symptoms would not be excluded). These individuals would be referred to individual mental health services.

### Measures

#### Usability study feedback questionnaire

Usability study participants and therapists were asked to rate each session on a 5-point scale from strongly disagree (1) to strongly agree (5) on the following aspects: usefulness, usability, desirability, value, accessibility, and credibility of each session, as well as their impressions of the online format, features, and general readiness for service-users (Orr et al., 2019). Example questions include, “This session provided information that helped me better understand my fear of cancer recurrence”, “I was able to follow along and understand this session with ease”, and “Overall, this session provided a deeper understanding of what fear of cancer recurrence is.”

#### Usability study exit interviews

Virtual semi-structured exit interviews were conducted with parents post-intervention by CH. Participating parents and therapists were asked questions regarding their expectations about the program, the general impact of the intervention on their FCR, and which segments they found helpful or unhelpful. Additionally, parents were asked about the administrative aspects of Parent-FORT, including timing and length, homework practice, and other supports. Finally, parents were asked about group cohesion, and whether they felt that any changes needed to be made to Parent-FORT, especially in relation to diversity factors (e.g., if gender or minority status was represented and welcomed in the intervention content).

#### Fidelity

Fidelity was rated using an adapted version of the fidelity checklist from our previous FORT studies (Lamarche et al., 2023; Lebel et al., 2014; Maheu et al., 2023; Tomei et al., 2018), which includes the following aspects: specific content and tasks, rated by completion (0 for no, 1 for yes); intervention-specific skills, rated from 0–2, (0 incomplete, 1 partially complete and 2 complete); and non-specific skills, also rated from 0–2. There were 21 specific content and tasks, 4 intervention-specific skills and 10 non-specific skills. Final adherence was rated out of 35 and converted to an overall percentage fidelity. Ratings of 80% or above across sessions were considered satisfactory.

#### Ethics

This study was granted an exemption from the University of Ottawa Research Ethics Board due to the quality improvement nature of the data collected from participants. It was assessed to present risks similar to a quality improvement initiative because participants were asked to provide their opinion of a clinical initiative to help with its continued improvement (e.g., is Parent-FORT ready, valuable, useful; are you satisfied with the content, etc.). In addition, no data on psychological outcomes (e.g., quality of life, FCR) were collected.

### Analyses

#### Quantitative analyses

Descriptive statistics were used to assess the results of the fidelity checklist and the feedback questionnaire completed after each session. Responses from parents and therapists were analyzed separately. Analyses were conducted using available data from parents who completed the intervention and missing responses were not imputed for parents who missed a single session.

#### Qualitative analyses

Interviews were audiotaped, transcribed verbatim, and managed using NVivo. Data were analyzed using inductive content analysis, in which codes are directly derived from the data (Hsieh and Shannon, 2005). Conventional content analysis was selected because of its applicability in qualitative evaluations of psychosocial interventions (Maheu et al., 2022). There were three phases to data analysis: (1) deriving discrete codes from the data, (2) congregation of related codes into categories, and (3) identification of patterns and relationships between the codes and categories (Graneheim et al., 2017). Last, categories were analyzed to develop themes. Themes are recurring concepts that emerge from analysis that typically represent underlying meaning between categories in content analysis and require a higher level of abstraction (Kleinheksel et al., 2020; Polit and Beck, 2017). Coding was conducted by a trained research assistant (CH). To facilitate trustworthiness, each transcript was read and independently double coded by the PI (SL). CH and SL met on a weekly basis to discuss the coding structure in the coding framework and assess for agreement and consensus. Trustworthiness of the findings were supported by (1) weekly debriefing sessions to reduce the risk of biased decisions; (2) the use of appropriate quotations; and (3) keeping an audit trail of self-reflexivity (Hsieh and Shannon, 2005; Miles and Huberman, 1994).

## Results

### Initial adaptation

The parent advisory board was given the option to adapt the original FORT manual, or the adapted manual for family caregivers of adult survivors of cancer (FC-FORT) (Lamarche et al., 2023, 2025). They unanimously chose to adapt the FC-FORT manual, as this content felt more relevant to their experience. Compared to FORT, FC-FORT additionally includes: (a) exercises aimed at addressing caregivers’ self-care and overcoming protective buffering; (b) suggestions to encourage having difficult conversations with loved ones regarding FCR; and (c) suggestions to optimize the use of loved ones’ health care teams. These revisions resulted in the addition of a seventh session in FC-FORT to incorporate this additional content.

The advisory board suggested several additional changes for the Parent-FORT intervention. Table 1 presents the content of each Parent-FORT session compared to the original FORT intervention. Major adaptations included adding exercises on how to talk to ones child when they have a new physical symptom; identity after the experience of childhood cancer; top three tools and wins to consolidate acquisition of skills as the group comes to an end; and providing resources for addressing siblings’ concerns. Wording around an exposure/worst case scenario exercise was also softened to “challenging case scenario”. Parents expressed that they got “stuck” on their most feared outcome, which led to a state of anxiety that disengaged them from the exercise. Moreover, many parents suggested that while they would feel comfortable to write this about their own cancer recurrence, they struggled to write this fear for their child as it felt like a form of manifesting their most feared outcome. Therefore, parents expressed that by modifying the wording to emphasize that this was one of many potentially challenging scenarios, they could engage with the exercise more effectively. In addition, a supplementary exercise discussing exposure versus manifesting was added to provide psychoeducation around exposure and reduce apprehension that writing out a challenging case scenario would increase the likelihood of a cancer recurrence. The advisory board also preferred the manual to act as a standalone guide that parents could reference post-treatment. As a result, more detailed instructions and psychoeducation about the scientific basis of the proposed exercises and tools were added. Furthermore, language was softened in terms of expectations around homework completion throughout the manual and wording was modified to reflect a parent–child relationship. Finally, the parent manual was professionally edited by a graphic designer to reflect a more modern aesthetic. See Table 2 for a complete description of modifications and their rationale.

**Table 1:** Comparison of changes between FORT and parent-FORT session.

Former sessions	Content	New sessions	Content
Session 1: Introduction to the group, learning new skills to deal with FCR (120 min)	Introduction by each participant with a focus on their experience with fear of cancer recurrence (FCR) Introduce ABC model of therapy, FCR model, cognitive restructuring and identify triggers Teach progressive muscular relaxation Homework: Practice progressive muscular relaxation daily; complete thought record and challenge maladaptive thinking about fear of cancer recurrence	Session 1: Introduction to the group, learning new skills to deal with fear of cancer recurrence (120 min)	Introduction by each participant with a focus on their experience with fear of cancer recurrence (FCR) Introduce ABC model of therapy, FCR model, cognitive restructuring and identify triggers Teach cognitive restructuring and self-care Homework: Complete thought record and practice self-care
Session 2: Providing information, increasing tolerance for uncertainty (120 min)	Prepare question for visit from health care professional who will be providing education about signs of recurrence in session 3 Help participants deal with the fact that uncertainty can never be completely eliminated Discuss ways of regaining a sense of control Teach the use of calming self-talk. Provide participants with relaxation files and instruct them to use calming self-talk phrases when appropriate. Homework: Listen to relaxation files every day; practice calming self-talk; complete thought record and challenge maladaptive thinking about fear of cancer recurrence and prepare questions for the nurse visit.	Session 2: Identifying your knowledge on cancer recurrence (120 min)	Discuss uncertainty and ways of regaining a sense of control Discuss the patient’s health care team Teach progressive muscle relaxation Homework: Complete session reflections, thought record and practice progressive muscle relaxation
Session 3: Building your coping skills (120 min)	Visit from a health care professional Increase tolerance for uncertainty by discussing acceptable level of worry Challenge faulty beliefs about benefits of worry Decrease maladaptive coping strategies Teach guided imagery Homework: Practice guided imagery daily; continue challenging faulty beliefs about benefits of worry; complete thought record adding a column for behaviors to monitor the kinds of coping strategies participants are adopting.	Session 3: Increasing your tolerance for uncertainty (120 min)	Discuss acceptable level of worry Challenge faulty beliefs about benefits of worry Explore how to productively focus worry Homework: Challenge faulty beliefs about benefits of worry. Practice calming self-talk and progressive muscle relaxation daily. Optionally, complete thought record.
Session 4: Getting deeper into underlying fears (120 min)	Provide psychoeducation about worry and the need for exposure to worst fears Promote emotion expression and confront specific fears that underlie FCR Write down worst fear scenario Teach mindfulness exercise Homework: Read worst case scenario every day and then do a self-care activity; Practice mindfulness exercise daily	Session 4: Building your coping skills (120 min)	Discuss unhelpful coping strategies Address communication difficulties Explore skills to address fear of cancer recurrence Teach guided imagery Homework: Having a conversation about FCR. Practice guided imagery daily; Practice progressive muscular relaxation; complete thought record with behavior
Session 5: Moving beyond specific fears (120 min)	Review exposure to worst case scenario exercise Discuss ways of coping with some of the feared outcomes Promote expression of feelings of demoralization Encourage participants to become re-engaged with important life goals, people, or activities they may have given up Discuss what meaning the future and planning now have for them Teach mindfulness exercise Homework: Write down goals and priorities for the future	Session 5: Getting deeper in the underlying fears (120 min)	Provide psychoeducation about worry and the need for exposure to worse fears Discuss difference between exposure to fear and manifesting feared scenario Promote emotion expression and confront specific fears that underlie FCR by writing down challenging case scenario Teach body scan meditation Discuss challenging case scenario and supports Homework: Read challenging case scenario daily. Practice self-care and body scan meditation exercise daily
Session 6: Review and conclusion (120 min)	Review all content covered Discuss future goals Setting new priorities Promote the expression of saying good-bye to the group and provide closure	Session 6: Moving beyond specific fears (120 min)	Review exposure to challenging case scenario exercise Discuss ways of coping with some of the feared outcomes Encourage participants to become re-engaged with important life goals, people, or activities Discuss what meaning the future and planning now have for them Teach mindfulness exercise Homework: Write down goals and priorities for the future, practice mindfulness exercise
		Session 7: Review and conclusion (120 min)	Review all content covered Discuss achievements and skills learned across sessions Promote the expression of saying goodbye to the group and provide closure Homework: Continue to practice self-care

**Table 2:** Summary of adaptations to Parent-FORT.

Categories of proposed changes	Final modifications
Addition of content	Added an emotions wheel to assist with identifying the different emotions parents might be feeling about their child’s cancer experience. Addition of key contacts chart to write down the names and important numbers for those who are now a part of the healthcare team. Addition of further “tips and tricks” that may be utilized during healthcare visits ex. Information about free healthcare apps that may be used to record conversations with health care professionals. Added practice 1: Session reflections to deepen understanding and key takeaways. Optional thought log added to the end of all session to be able to track across sessions. Questions added to the end of worry questionnaire to further prompt parents to reflect on their scores/responses. Additional exercise added, “Coping when your child has a new symptom” to further explain helpful and unhelpful ways to respond to this situation. Additional “Exposure vs. Manifesting” exercise added to highlight that thinking about something does not make it more likely to happen. Additional exercise added, “Unpacking your challenging case scenario” to further explore tools and resources available to parents to help them face their challenging scenario. End of therapy practice added: “Practicing your self-care.” Encourage parents to continue to practice self-care and set aside time as they have for this therapy group.
Removal of content	Removed visit from healthcare professional in Session 3. For Calming Self-Talk exercise in session 3, only one example trigger noted instead of several. Session 6 session reflection questions shortened. First video removed from Session 4 exercise, “video about communicating during difficult times”. Parent advisory felt it did not resonate and was too abrupt as it talked about grief.
Substitution of content	Exercise changed from “Regaining control over our lives” exercise, which involved ranking priorities and filling areas on a pie chart, to “Productive vs. unproductive worry”, which involves following the tree diagram to determine whether worries are “today” problems, or “future/hypothetical” problems. Session 7 Exercise changed from balancing my life exercise to “My top 3 tools and wins”. Focus on how far parents have come and concretely reflect on a summary of tools that work for them. Avoidance example changed from fear of dogs to fear of car accident based on therapist and parent feedback during usability. Exercise changed from “communicating with [cancer survivor] about fear of cancer recurrence” to communicating with others, as parents felt it was important for them to be able to share their concerns with others, not their child.
Addition of information/psychoeducation	Added a group rules and reminders page to discuss confidentiality and supports/resources parents can use if they become overwhelmed. Added introductions to several exercises to explain its basis/why it may be helpful. Added psychoeducation, “The worry balancing act”. Includes description of finding a good, constructive amount of worry based on the Yerkes-Dodson Law. Added note that parents can revisit the workbook at any time. Additionally, acknowledged all the work parents have done during this therapy. Additional examples added to exercises (ex. additional unhelpful coping strategies, calming self-talk phrases, etc.) to provide ideas to parents while working through sessions.
Language modifications	Overview and goals of sessions pages modified to reflect updated content. Changed the wording throughout to reflect parents/caregivers/guardians. Exercise titles changed to better reflect content. Language softened throughout ex. Changed post session exercises from “Homework” to “Practice”; Worst case scenario exercise (previously called “Identify Your Most Challenging Fear”) changed to “Challenging Case Scenario” to make content more sensitive and approachable. Introductory paragraphs/questions modified to include additional supportive elements and resonate more effectively with parents. Examples changed to be relevant to parents & children. For example, modified communication difficulties section to reflect parent–child relationship. Challenging case scenario changed from spouse to child. Name of doctor and child changed. Scenario changed from stopping treatment to deciding what to do next.
Formatting	Worked with a graphic designer to change the colours and icons so that they reflect something more similar to the caregiver workbook (modern, streamlined feel). Changed ABC models to make them more applicable to parents ex. Added an additional example to ensure different childhood cancers/symptoms are reflected in the model. Space added to brainstorm self-care activities. Thought log changed to landscape to provide more space to write. Session 6 “Who am I now?” exercise format changed so that it is columns of before, during, and after cancer, rather than listed questions. More about the reflection and planning piece, and helping parents to transition, rather than strictly focusing on FCR. Session 4 avoidance vs. exposure graphs formatted to better coincide with descriptive paragraphs.
Technical features	Changed offering of therapy to 1-month post-treatment rather than 1-year post-treatment to increase accessibility and ensure clinical relevance of materials.
Resources	Added additional resources to end of workbook. Made resources parent & child specific, added parent support groups, broader variety of cancer-related topics, and added province-specific resources.

With respect to coding changes per FRAME, initial agreement between coders was 92.5% and discrepancies were resolved through discussion (see Figure 1 for the FRAME key modification considerations). All modifications (100%) occurred pre-implementation, and thus all adaptations (100%) were planned/proactive in nature. Almost all modification decisions (99%) were made collaboratively with the research team and the parent advisory board. One modification was made based on therapist and parent feedback during the usability study. The parent advisory board proposed all other suggested changes, which the research team then evaluated based on clinical judgment, theoretical knowledge, and fidelity to the intervention before being implemented. The goals of Parent-FORT modifications included increasing parent satisfaction (41%), improving fit with recipients (33%), increasing retention (i.e., changes that increase participants’ willingness to engage with a full dose of therapy; 13%), improving effectiveness of the therapy (11%), and increasing reach or engagement (1%). Nearly all modifications (99%) were considered content modifications. The one exception was the decision to offer Parent-FORT to parents whose children were 1-month post-treatment rather than 1-year post-treatment. This was considered an implementation activity, as it adapted our strategy used to implement the intervention (Wiltsey Stirman et al., 2019). This decision was made based on feedback from the parent advisory board and subsequent assurance during the usability study that accessing Parent-FORT more quickly post-treatment would increase its relevance and reach parents most in need of the therapy. All modifications (100%) were made to address the target intervention group (i.e., parents). Most content modifications (89%) were coded as tweaking or tailoring, which is considered a change to the intervention that preserves the major intervention principles and techniques, but adapts the intervention’s language, develops an updated version of exercises, or makes the intervention more culture or population specific. Other modifications included adding modules (7%) (e.g., additional exercise added, “Coping when your child has a new symptom”) and substituting (4%) (e.g., “Balancing my life” exercise in final session replaced with “My top 3 tools and wins” exercise). The reason for all modifications (100%) was at the organizational level to better align with the goal and purpose of the therapy. For a comprehensive list of all changes, please see Table 2.

### Usability study

After the intervention was adapted, a usability study of Parent-FORT was conducted between October and December 2024.

#### Participation

**Figure 2:**
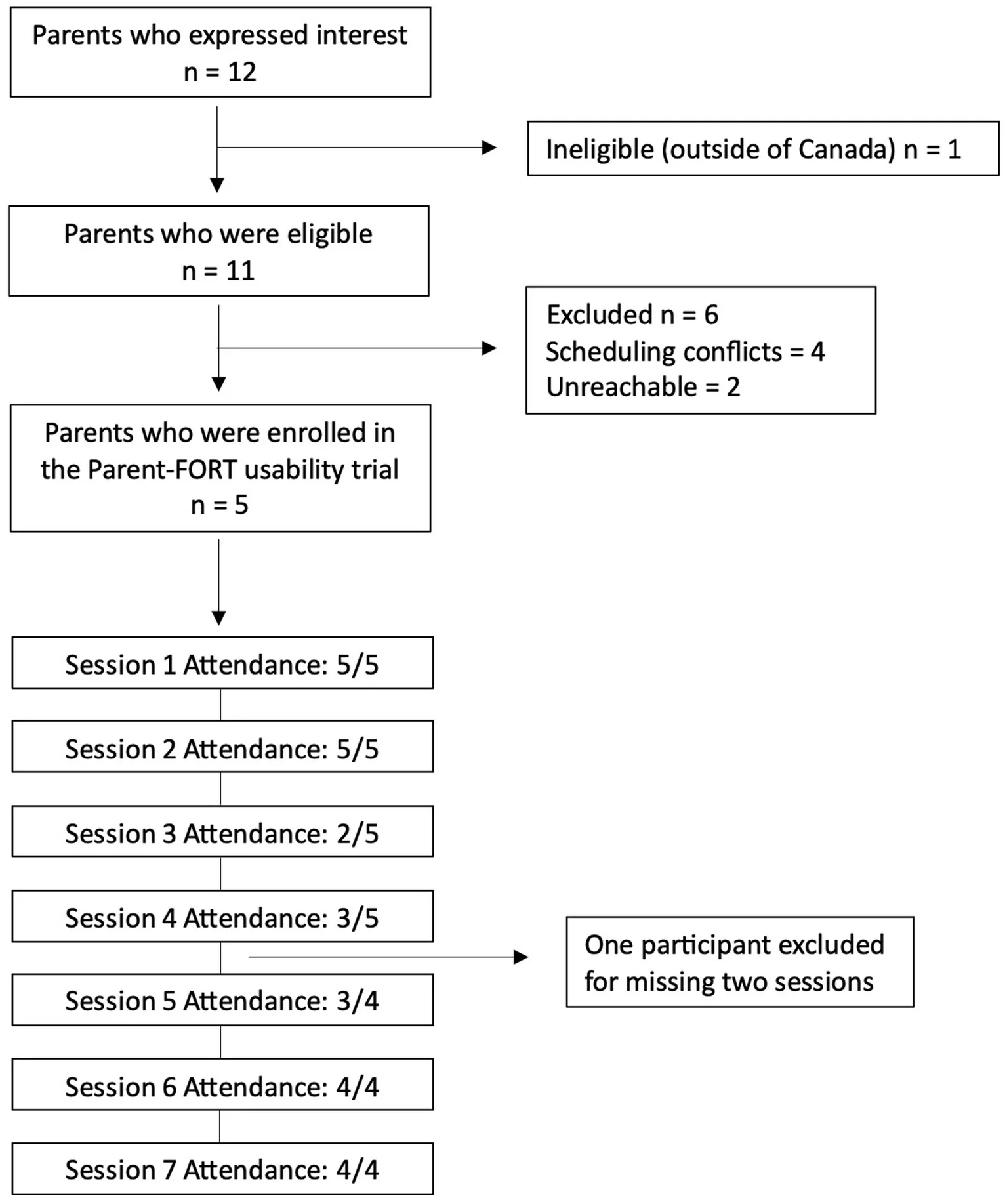
Flow diagram of study sample.

Two therapists took part in the usability study as co-facilitators. Twelve parents expressed interest in participating in the study, eleven were deemed eligible, and five parents were enrolled. One parent was excluded after session 4 due to an unexpected change in availability which resulted in missing two sessions and was invited to start again with a subsequent group. The four remaining parents attended, on average, 6 of the 7 sessions. A flow chart of participation including reasons for exclusion can be found in Figure 2. Participant demographics can be found in Table 3. Parent participants who completed the intervention (100% mothers) were living in Alberta (50%) or Ontario (50%). Participants’ children were diagnosed with cancer between the ages of 2–16, and had been off treatment for 1–8 years.

**Table 3:** Sample characteristics of parents who completed the usability study (*N* = 4).

Variable	Values
Age (years), mean (SD)	45.0 (5.3)
Age of child at diagnosis, mean (SD)	6.5 (6.5)
Time since completion of treatment, mean (SD)	4.3 (3.0)
Ethnicity, *n* (%)	
White	3 (75%)
South Asian	1 (25%)
Province, *n* (%)	
Alberta	2 (50%)
Ontario	2 (50%)
Marital status, *n* (%)	
Married	3 (75%)
Common law	1 (25%)
Employment status, *n* (%)	
Full time	1 (25%)
Student	1 (25%)
Unemployed due to illness	2 (50%)
Level of education, *n* (%)	
University Degree	4 (100%)
Annual income level, *n* (%)	
$81,000–$100,000	2 (50%)
Greater than $100,000	1 (25%)
Undisclosed	1 (25%)
Child’s cancer type, *n* (%)	
High risk neuroblastoma	1 (25%)
Stage 3 Hodgkin’s lymphoma	1 (25%)
Medulloblastoma	1 (25%)
Undisclosed	1 (25%)

#### Fidelity

All sessions had a fidelity rating of 80% or above, with an average therapist administration fidelity of 90%.

#### Adaptations and changes

Only two additional modifications were made as a result of the usability study. These included: changing the exclusion criteria from children being 12-months post treatment to one-month post treatment and modifying one example of avoidance to better align with parent preferences.

#### Feedback questionnaires

Only data from the four parents who completed the study were included in final analyses. Across all seven sessions, 81% of surveys were completed by parents who attended the session, and 100% of surveys were completed by therapists. Overall, parents and therapists found Parent-FORT to be acceptable, and exercises were well received by participants. The mean satisfaction rating for parents and therapists combined was “Extremely Satisfied.” Parent-FORT was rated as “Very Ready” by parents and “Extremely Ready” by therapists. Generally, parents and therapists found that Parent-FORT was useful, usable, desirable, accessible and valuable (see Tables 4, 5, respectively). They reported being very satisfied with the intervention and rated it as very ready for future participants. Overall session ratings were generally high across both parents and therapists.

**Table 4:** Parents’ session feedback questionnaires (mean scores) (*N* = 4).

Feedback category	Scale	Session 1	Session 2^†^	Session 3^‡^	Session 4^‡^	Session 5	Session 6	Session 7	Overall
Useful	1–5	3.6	3.7	4.0	4.4	4.0	4.3	4.0	4.0
Usable	1–5	4.8	4.0	4.7	4.5	4.3	4.3	4.0	4.4
Desirable	1–5	4.8	4.0	4.7	4.3	4.0	4.3	4.0	4.3
Accessible	1–5	4.8	4.3	4.8	4.0	4.3	4.7	4.0	4.4
Features	1–5	4.2	4.0	4.3	4.1	4.0	4.6	4.3	4.2
Valuable	1–5	4.1	4.2	4.2	4.4	3.9	4.3	4.7	4.3
Readiness	1–5	3.5	3.3	4.3	4.0	3.7	4.0	4.0	3.8
Overall satisfaction	1–5	4.0	3.7	4.3	4.3	3.7	4.3	5.0	4.2
Satisfaction with content^a^	1–3	2.8	2.9	3.0	2.7	2.8	3.0	3.0	2.9
Satisfactory amount of information and tools^a^	1–3	2.5	2.3	3.0	2.6	2.7	2.7	3.0	2.7
Total score	10–46	39.1	36.4	41.3	39.3	37.4	40.5	40.0	39.1

Higher scores reflect more positive responses.

All scores are on a 5-point Likert Scale (“strongly disagree” to “strongly agree”; “not at all ready” to “extremely ready”; “not at all satisfied” to “extremely satisfied”). ^a^Scores are on a 3-point Likert Scale (“no”, “maybe”, “yes”), with the highest possible score being a 3. ^†^Feedback scores from session 2 reflect *N* = 2. ^‡^Feedback scores from sessions 3 and 4 reflect *N* = 3.

**Table 5:** Therapists’ session feedback questionnaires (mean scores) (*N* = 2).

Feedback category	Scale	Session 1	Session 2	Session 3	Session 4	Session 5	Session 6	Session 7	Overall
Useful	1–5	5.0	5.0	5.0	5.0	5.0	5.0	4.3	4.8
Usable	1–5	5.0	5.0	5.0	4.5	4.8	5.0	5.0	4.9
Desirable	1–5	4.5	5.0	5.0	4.5	5.0	5.0	4.5	4.8
Accessible	1–5	4.5	5.0	4.5	4.8	5.0	5.0	5.0	4.8
Features	1–5	4.5	4.3	5.0	5.0	4.7	4.7	4.5	4.7
Valuable	1–5	4.8	4.8	4.8	4.8	4.8	4.8	4.5	4.8
Readiness	1–5	4.5	5.0	4.5	4.0	4.5	5.0	5.0	4.6
Overall satisfaction	1–5	4.5	5.0	4.5	4.5	5.0	5.0	5.0	4.8
Satisfaction with content^a^	1–3	3.0	3.0	3.0	2.7	3.0	2.7	3.0	2.9
Satisfactory amount of information and tools^a^	1–3	2.8	2.8	3.0	3.0	3.0	3.0	3.0	2.9
Total score	10–46	41.3	43.9	43.3	41.8	43.5	44.2	42.8	43.0

Higher scores reflect more positive responses.

All scores are on a 5-point Likert Scale (“strongly disagree” to “strongly agree”; “not at all ready” to “extremely ready”; “not at all satisfied” to “extremely satisfied”). ^a^Scores are on a 3-point Likert Scale (“no”, “maybe”, “yes”), with the highest possible score being a 3.

#### Exit interviews

All participants were invited to take part in the exit interviews; three participants and both therapists consented. Four key categories were identified through content analysis: connectedness of the group; general format; satisfaction with the content and exercises; and overall impact.

##### Connectedness of the group

Participants appreciated the size of the group as they felt it allowed them to open up to others more easily and better connect with the other parents. There was also consensus that parents were highly supportive of one another early on in the group:

*“I think at the beginning everyone was in their own rectangle on the screen, you know what I mean? But by the end, like even by the second session there was people crying along with other people. There were people, you know, clapping for other people. There were people giving thumbs up. And so I just felt connected.” (P3)*

All participants emphasized the importance of sharing their experience with others who understood their situation, and that the connectedness that emerged as a result of sharing their story helped them to challenge some of their thoughts and fears around cancer recurrence:

*“It was just nice to have some kind of validation, I guess would be the right word that it’s normal for this kind of thing [FCR]. Cause sometimes I felt like, am I overreacting? Am I just being Chicken Little? But it seemed like everybody…we were all in different stages post treatment, and everyone kind of felt the same way. And it was nice to feel like not an island.” (P5)*

##### General format

Parents and therapists both felt that the length of the sessions were appropriate, as well as the length of the overall therapy. They also reported that they appreciated having the sessions virtually, as it afforded them more flexibility, as well as accessibility, without affecting their ability to connect to the other participants:

*“I thought it was great, it was convenient. I think by now we are all used to this, so it did not bother me at all.” (P1)*

Participants also identified that the virtual format provided them with a sense of comfort to be able to log on from home and feel healthy separation from the group when needed:

*“I think it definitely helped me to be a little bit more outspoken than I normally would if it were in person. The one [group] I did go to was in person, and I did not talk the entire time because it was too many eyes and just too much. Whereas online, even though you are having a connection, there’s still a little bit of disconnect where it’s not necessarily there if you are in person, that disconnect. So I thought the online part was nice.” (P5)*

The timing of the group worked well for most parents. However, there was recognition that a midday group may not work for parents who are unable to take time off of work. Furthermore, the most appropriate time for parents may fluctuate depending on the age of their child, as well as parent’s location within Canada and feasibility across time zones.

One parent expressed that while the therapy sessions were helpful, they may have benefited more greatly if the sessions were offered closer to the cessation of treatment:

*“A lot of the things that were covered were things that I already knew about. There’s a lot of cognitive behavioral therapy, which I was already very familiar with…But had I done this, you know, a few years ago when my daughter had first finished treatment, I think that would have moved it a little bit closer to ten.” (P1)*

##### Satisfaction with the content and exercises

Parents and therapists reported feeling very satisfied with Parent-FORT (i.e., workbook, content, coping strategies). Parents highlighted a range of exercises that were most helpful to them (e.g., thought log, unpacking their challenging case scenario, guided meditation):

*“Like, you could use your breathing. You could use the guided meditation and then [note] where you were afterwards, I felt that was really, really helpful.” (P3)*

Some parents noted that certain exercises or homework practices were less useful for them. However, there were no elements of Parent-FORT that parents found unhelpful or that they felt should be removed.

Parents had mixed feelings about the exposure exercise in session 5. Most parents noted that it was challenging, but they appreciated the opportunity to explore their fears around their child’s cancer coming back and found it helpful to have the guidance of the therapists. However, one parent noted that they would appreciate more notice and explanation around what the exercise would entail so that parents are emotionally prepared to engage with the exercise:

*“I would say the main part of the program that was referred to as the crux or the peak or whatever, just more of a prep or a heads up prior. I do not know, I just felt like, to find out in the session that that’s what the plan was, was a lot. So just more of a heads up that it’s going to be really heavy, it’s not going to be easy, that kind of thing.” (P1)*

##### Overall impact

When asked during the exit interview how important the group had been for them [(on a continuum from 0 Not important) to 10 (Extremely important)], parents rated, on average, the importance of Parent-FORT as a 7. In addition, all participants indicated that they would recommend Parent-FORT to others:

*“I would, just because it’s one of the few programs out there that even exists. And I truly believe it is helpful for parents whose kids are just finishing treatment.” (P1)*

All parents expressed that the therapy was beneficial to them. They were better able to identify their triggers and avoidance strategies, as well as utilize different exercises to manage these. Qualitatively, one parent noted that they have found they are better able to cope with their fear of cancer recurrence after participating in Parent-FORT:

*“Like even now, the past few weeks of not having [the group] every week, like [parent’s child] is sick today with a fever and her leg is hurting and I did not immediately jump to, oh my God, she’s going to die. Because usually it would be pretty fast, like 0 to 60 for me. And I do not know if it was just internalizing the things that I learned or just knowing that it’s normal in our population of people who have had children with cancer. But it’s definitely benefited me. Like normally I’d be rocking back and forth crying at this point if she had a fever and was sick. But I’m managing it.” (P5)*

Outside of their FCR, parents found that the skills they developed through the Parent- FORT program have given them confidence and reassurance that they can manage stressful situations more generally:

*“I was not in control of [participant’s child] having cancer, right? It wasn’t because of something I did where he got cancer. But being able to control any aspect, or at least have the tools in order to work through any aspect of your life that you possibly can is huge, and that’s what this course gave me.” (P3)*

Most parents expressed that the therapy has continued to have an impact on their life after ending the group:

*“I was left kind of alone with my thoughts before this. Whereas now, I’ve referenced our workbook a number of times already, right? And I’ve gone back and not even with cancer, but just tricky situations that I’ve gone back.” (P3)*

##### Readiness for pilot testing

Following completion of the usability study, session ratings and qualitative feedback was shared with the broader study team. As reported above, no session received low overall ratings and suggested revisions were limited to minor changes. As such, consistent with our criteria for pursuing additional usability testing, the research team determined that additional usability testing was not warranted and that the intervention was ready for pilot feasibility testing.

## Discussion

The purpose of this study was to adapt the content of FORT for parents of survivors of childhood cancer and test its usability among a group of parents. To do so, we sought to meaningfully and collaboratively engage parents with lived experience throughout the adaptation and usability study process. Key changes from the adaptation phase included adding exercises about responding to children’s physical symptoms and exploring parents’ identity after the experience of childhood cancer. Minor changes included softening language, modifying wording to reflect a parent–child relationship, and increasing psychoeducation. Additional descriptors were also added to several of the exercises to allow the manual to act as more of a standalone guide that parents could reference after the conclusion of the Parent-FORT group. Feedback from parents and therapists in the usability study indicated that the program was useful, usable, desirable, accessible, and valuable, with minimal requested changes, indicating readiness for use in a pilot feasibility trial. As a result of the usability study, eligibility criteria was revised from one-year post-treatment to one-month post-treatment to increase accessibility and clinical relevance. Of all modifications made, nearly three-quarters reflected minor tweaks to increase parent satisfaction and improve overall fit with recipients. All core elements of FORT were preserved and Parent-FORT therefore maintained fidelity as a group-based cognitive-existential therapy aimed at addressing FCR. Participants endorsed that they would recommend Parent-FORT to other parents and found its exercises to be helpful in dealing with FCR and other stressors.

Throughout the adaptation process as well as the group sessions, parents expressed multifaceted concerns, including challenges with communicating FCR to loved ones, explaining cancer to other children/siblings, parental guilt connected to time away from other children, the possibility of secondary cancers, and late effects. While not all exclusively related to FCR, all concerns identified were associated with their child’s cancer history. These additional needs were considered, while recognizing that it was important to ensure the intervention maintained its validity and original purpose. Thus, while this intervention focused on predominantly FCR, there was an appreciation that Parent-FORT seeks to address worry about health effects broadly.

An additional topic raised by parents was about manifestation. Parents voiced concern that writing or talking about their most feared outcomes in relation to their survivor child could manifest these fears into reality. This concern has important implications for parent–child communication surrounding cancer, particularly for parents who may limit conversations due to concerns that it would increase the likelihood of a recurrence. To address this, we added session content aimed at addressing the difference between manifesting and exposure work. Further emphasis was also placed on communicating with children about difficult topics beyond FCR, like presenting with a new symptom, to highlight healthy modeling of emotions and coping. Overall, approaching session activities and discussions with sensitivity and care was crucial to ensuring responsiveness and engagement with difficult topics, particularly with the challenging case scenario exercise.

Notably, some anticipated themes from the literature, like giving young survivors more control of their medical care, were not brought up by the advisory board or participants in the usability study. This theme may carry more relevance depending on the age of a parent’s child and is an important consideration as key topics may shift as children move through development (Tutelman and Heathcote, 2020).

Aside from the content of the intervention, findings from our qualitative interviews indicated that the group therapy format of Parent-FORT was especially valuable for participants. Parents described significant benefit from connecting with others who shared similar fears about their children’s health and feeling understood by both other participants and the group therapists. The group modality also offers meaningful clinical advantages, as it supports scalability and feasibility within healthcare and community settings that often face limited resources (Valentine et al., 2023).

Finally, as noted, the most significant change made during the usability phase was the change in eligibility criteria from one-year post completion of treatment to one-month post-completion. Qualitative findings from the usability study highlighted the critical period parents face at the end of treatment. As one parent noted, several themes were familiar to her prior to her participation in the group sessions and may have resonated more strongly if the therapy had been available to her at the end of her child’s treatment. Thus, offering Parent-FORT to parents and caregivers as they transition out of active treatment may improve communication between parents and their children, their families, and their child’s healthcare team, and reduce the likelihood of prolonged FCR.

### Reflections on involvement: The perspective of parent partners

The adaptation of the Parent-FORT intervention was conducted using an iterative, collaborative approach that integrated parent partners throughout all phases of the project. Parent advisory board co-chairs were embedded within the research team and contributed through their lived experience and expertise. Their ongoing involvement supported continuity across phases of adaptation and facilitated alignment between the advisory process and intervention adaptation. The parent co-chairs also reviewed the final intervention materials to ensure consistency with the parent advisory board’s feedback and the intended goals of the adaptation.

Parent advisory board participants were recruited to reflect diversity in caregiving experiences, geography, and personal backgrounds. Feedback was obtained through structured conversations facilitated by the parent co-chairs. Engagement strategies, including roundtable-style feedback and regularly scheduled meetings, were used to support balanced participation. Moreover, equitable participation and psychological safety were emphasized to support open discussion and the incorporation of diverse lived experiences into adaptation decisions. The parent advisory board contributed to multiple aspects of the intervention, including language, content relevance, accessibility, and visual presentation.

Input provided by the parent co-chairs and the advisory board enabled substantial refinement of the intervention prior to usability testing and may have contributed to the high readiness ratings reported by parents and therapists, as well as the limited number of substantive modifications required following usability evaluation. Parent-FORT had an overall parent satisfaction score of 4.1/5, an overall readiness for future users score of 3.9/5, and a rating of “extremely ready” from therapists. Only two minor modifications were required following usability testing, including broadening eligibility from 12 months to 1-month post-treatment and revising one avoidance example used by therapists. High ratings of usefulness and value, together with qualitative feedback indicating strong satisfaction with the content and exercises, further support the acceptability of the adapted intervention. Participants reported strong group connectedness, sustained engagement, consistent attendance, and appreciation for shared experiences, suggesting that facilitation strategies emphasizing inclusivity and psychological safety supported both the adaptation process and later intervention delivery.

Some considerations for future adaptation work were identified. Future studies may consider incorporating smaller breakout sessions between larger group meetings, allowing quieter participants more space to contribute. Additional time for review, for example, having written feedback submitted in advance and dedicated reading time during meetings, could allow for deeper exploration of content. More frequent updates between sessions may also strengthen engagement and continuity. The incorporation of anonymous survey or polling tools alongside group discussion may also enhance future adaptation processes by creating additional opportunities for candid feedback. Additionally, greater inclusion of fathers would improve representativeness. These considerations highlight opportunities to further strengthen future adaptation initiatives while supporting the overall findings of this study.

Collectively, the findings suggest that embedding parent partners within the research team, combined with a structured and facilitated advisory group process, improves efficiency, relevance, and acceptability of intervention adaptations and may reduce the extent of revisions required during later stages of usability testing.

### Limitations and future considerations

With respect to the usability study, we note that the session feedback questionnaires have not been explicitly validated in this population (but rather had been used in our previous adaptation of FORT to family caregivers) and thus should be interpreted within the necessary exploratory context of this work. Our usability study sample size was also smaller than intended, in part due to logistical challenges. Specifically, there were several parents who were interested in participating but were unable to do so due to scheduling challenges. Future feasibility testing of Parent-FORT and other nationwide virtual interventions may consider addressing this barrier by having therapists from several time zones to accommodate parent schedules.

Based on feedback from parents, the decision was made to increase eligibility for the program to include those who had more recently completed treatment, which may expand the pool of eligible parents for future studies. That said, it is possible that our results may have differed for those nearer off treatment. Parents also highlighted a gap in available supports for siblings, suggesting an important direction for future resource development and family-centered survivorship care.

We acknowledge that our both our adaptation team and usability study sample were predominately white mothers. As such, future efforts on Parent-FORT will need to prioritize recruitment of fathers and culturally diverse parents. This could include using culturally inclusive recruitment materials, father-directed language and images, and strategic partnership with community organizations. Future work will continue to prioritize the involvement of parents with lived experience, who may also have ideas about how to increase engagement with fathers and culturally diverse populations. Parent partners will remain embedded within the broader research team and contribute to all aspects of future work from study design, to recruitment, interpretation of results, and knowledge translation and mobilization efforts.

## Conclusion

Parent-FORT reflects the first intervention designed specifically to address FCR experienced by parents of survivors of childhood cancer, almost half of whom report clinically significant FCR (Webb et al., 2023). The original FORT intervention was successfully adapted for parents of survivors of childhood cancer (Parent-FORT) through co-design. The inclusion of parents with lived experience as equal partners in the adaptation was an essential method of this work. Results of the current project indicate preliminary evidence of usability and acceptability, supporting readiness for a future feasibility pilot study of Parent-FORT.

## Data Availability

The original contributions presented in the study are included in the article/supplementary material, further inquiries can be directed to the corresponding author.
